# The Feeding-swallowing Impact Survey: Reference Values from a UK Sample of Parents of Children Without a Known or Suspected Feeding Disorder

**DOI:** 10.1007/s00455-025-10845-z

**Published:** 2025-06-05

**Authors:** Alexandra Stewart, E. Van Hoeve, A. Mustafa, M. A. Lefton-Greif, D. Ridout, C. H. Smith

**Affiliations:** 1 Department of Language and Cognition, University College London, London, UK; 2 Department of Speech and Language Therapy, Great Ormond Street Hospital, London, UK; 3 Departments of Pediatrics, Otolaryngology-Head and Neck Surgery, Physical Medicine & Rehabilitation, Johns Hopkins University School of Medicine, Baltimore, USA; 4 UCL Great Ormond Street Institute of Child Health, London, UK

**Keywords:** Deglutition, Feeding difficulties, Pediatric feeding disorder, Outcome, Quality of life, Caregiver

## Abstract

The feeding swallowing impact survey (FS-IS) is an 18-question, 3 subscale, validated tool measuring parent/caregiver impact of children’s eating, drinking and swallowing difficulties. This study aimed to establish reference values from parents of children without known or suspected paediatric feeding disorder for the FS-IS. Parents of children aged 6 months-11 years were invited to complete an anonymous, online questionnaire. The Pedi-EAT-10 was used to screen for pediatric feeding disorder. For the FS-IS average and cumulative scores were calculated for the whole scale and subscales. Percentile rankings were calculated for the cumulative whole scale score. The relationship between variables was explored using Spearman’s correlation and 75th centile quantile regression. The final sample consisted of 904 parents. Median (IQR) whole scale score was 1.11 (1.06, 1.22), from a possible range of 1-5.. Percentile rank scores were: 5th = 18, 25th = 19, 50th percentile = 20, 75th = 22 and 95th = 29, from a possible range of 18-70. Parent perception of a feeding difficulty was the factor associated with greatest score increase. These data provide UK reference data for the FS-IS, facilitating its use in the evaluation of parental impact of feeding difficulties, a key component of holistic, family-centred care. Feeding-related QOL is largely unaffected in caregivers of children without a diagnosed or suspected pediatric feeding disorder.

**Supplementary Information:**

The online version contains supplementary material available at 10.1007/s00455-025-10845-z.

## Introduction

Caring for a child with a paediatric feeding disorder (PFD), whether rooted in medical, feeding, nutritional or psychosocial domains, can result in reduced quality of life [1]. Studies have demonstrated high levels of parental anxiety, stress, isolation, limits to social activity and ability to work, as well as negative impacts to family relations [2–6]. To measure these feeding-related QOL impacts, Lefton-Greif and colleagues developed the feeding/swallowing impact survey (FS-IS) [7]. Initial validation for the 18-item FS-IS was undertaken with 164 children (median age 14 months, IQR 7–35 months) attending a tertiary feeding/swallowing clinic. The FS-IS has undergone cross-cultural adaptation and translation into a number of languages [8–10]. It has been utilised in different patient groups including eosinophilic esophagitis [11], oesophageal atresia [12], Down Syndrome [13], laryngeal cleft [14] and cleft palate [15].

Problematic mealtime behaviours are also commonly reported by parents typically developing, healthy children [1, 16]. Behaviours such as “picky eating”, food refusal (particularly fruit and vegetables), eating snacks instead of meals and a dislike of trying new foods were described by 10–60% of parents of children up to 11 years of age [17–21]. These presentations have been associated with parental concern, frustration, guilt and stress [22–24] and been found to negatively impact on family relations [19].

The FS-IS is missing reference data from a population of children *without* diagnosed or suspected feeding and swallowing disorders but for whom some suboptimal eating and drinking behaviours may exist. Given the reported impact of problematic but not disordered feeding or swallowing on parental QOL, the absence of reference data restricts the ability to fully understand the significance of FS-IS scores obtained in those *with* a diagnosed feeding disorder, thereby limiting its use in clinical and research practice. The aim of this study was:

1. To generate reference values for FS-IS subscale and total scores from parents of children aged 6 months-11 years without a diagnosed or suspected Pediatric Feeding Disorder.

The following specific objectives were set:

(i) To determine average values and spread of FS-IS subscale and total scores.

(ii) To generate percentile rankings for the total FS-IS score.

(iii) To identify variables associated with FS-IS total score.

2. Methods

This study was a cross-sectional observational study using an online questionnaire method. Study data were collected and managed using REDCap electronic data capture tools hosted at the authors institution. Data were collected anonymously. Ethical approval was granted at the participating institution.

### Materials

#### Questionnaire Design

The questionnaire was piloted by six parents for readability and ease of completion. Minor revisions to wording were incorporated into the final questionnaire (supplementary material 1). The final questionnaire consisted of: a participant information sheet and electronic consent, demographic and brief medical history, a screen for swallow/feeding dysfunction (Pedi-EAT-10) [25, 26] and the FS-IS [7].

#### Demographic and Medical History

Participants provided demographic and medical history data (supplementary material 1). Child age was collected as whole numbers representing completed years (i.e. 6–11 months, 1 year, 2 years etc.). If parents had more than one child aged 6 months-11 years, they were asked to answer the questions with one of their children in mind.

#### Feeding-Swallowing Impact Survey (FS-IS)

The FS-IS is an 18-item parent-reported tool for assessing feeding-related quality of life (i.e., the impact of a child’s eating and drinking on the parent/family) [7]. Using a one-month recall period, participants are asked “As a result of your child’s feeding/swallowing problems, how often have you had problems.?”. Participants used a ranked order scale ranging from 1 to 5, with 1 representing “never” and 5 representing “almost always”. Minor wording modifications were made to tailor the scale to parents of children *without* known feeding/swallowing difficulties. For example, the word “problem” was placed in brackets, “It is hard to feed my child because family members or professionals have different opinions about how to take care of my child’s feeding/swallowing (problems)”. There are three subscales: Activity (5 items), Worry (7 items), Feeding (6 items). Scores are summed and divided by the number of items to generate subscale and whole scale scores out of 5.

The FS-IS demonstrated good internal reliability (Cronbach’s alpha above 0.7 for total score and subscale scores). Construct validity was demonstrated with moderate correlation with the PEDS-QL Family Impact Module (Pearson correlation *r* = 0.23–0.62).

#### Pedi-EAT-10

The Pedi-EAT-10 is a 10-item parent-reported screening tool for swallowing difficulties with excellent intra-rater reliability and good internal consistency [25]. Good concurrent validity with the penetration-aspiration scale has been demonstrated [25, 27]. The Pedi-EAT-10 was found to discriminate between infants (mean age 7 months) with normal and abnormal videofluoroscopy, those receiving thickened liquids and those receiving tube-feeding [26]. Using a one-month recall period, participants rate each question using a 5-point Likert scale (0 = no problem, 4 = severe problem). A summative score is generated. A summative score of four or more indicates potential feeding or swallow difficulties [25].

#### Recruitment

A volunteer sample was recruited using a multi-modal recruitment strategy:

1. *Online advertising on social media*. Links to the questionnaire were shared by the research team on Twitter (now “X”), Instagram, Facebook, Reddit, Nextdoor, Mumsnet, Tiktok and Whatsapp.

2. *Email to targeted services*. To engage with groups typically underrepresented in research – (i.e., those living in areas of social deprivation, those of black or minority ethnicity, and fathers) links to the questionnaire were sent to selected elementary schools, childcare settings and places of worship with a request to share with their communities.

3. *Snowball sampling*. Participants were encouraged to share the link with their own social networks, for example sharing on Whatsapp groups or social media sites [28].

#### Participants

Parents or legal guardians of a child aged 6 months-11 years with no known feeding or swallowing difficulties were included. Parents were living in the UK and over the age of 18 years. Participants scoring four or more on the Pedi-EAT-10 were determined to be at risk of feeding/swallowing difficulties and excluded from the sample.

#### Data Analysis

Questionnaire data were downloaded from REDCap in .sav format and imported into SPSS version 29 (released 2022) for analysis. Data were tested for normality using histograms and Kolmogorov-Smirnov tests [29]. FS-IS scores were positively skewed, as demonstrated by visual inspection of the data and a significant Kolmogorov-Smirnov test *(p* = < 0.001). Therefore, data are reported using medians and non-parametric statistical tests. Mean scores are also provided as this scoring system was used during initial development and validation of the tool [7].

Given the limited spread of data when scored using summed averages, cumulative averages were also calculated. Cumulative scores were determined to be easier to meaningfully interpret differences in score and were therefore used for the remainder of the analysis. The two scoring methods are defined as:

1. Average score = summative score divided by the number of questions
2. Cumulative score = summative score

Categorical data were described using frequency and percentage.

Group differences in FS-IS total scores according to demographic variables were assessed using Mann-Whitney U tests for comparisons between 2 groups and Kruskall-Wallis test for variables with three or more groups with Bonferroni adjustment for multiple comparisons. These results were used to develop a regression model to assess factors impacting on score variability. As whole scale cumulative scores were highly positively skewed, we used quantile regression to model the relationship between demographic and clinical factors with the 75th centile whole scale cumulative feeding scores. We chose to model the 75th centile because we were interested in understanding which factors are associated with a high total score. Significance levels were set at 0.05.

To generate reference values, whole scale cumulative scores were used to calculate descriptive centiles (5th, 25th, 50th, 75th and 95th) for the whole cohort and children under 2 years and over 2 years. These age groups were selected as it would be anticipated that most children would be eating family foods by the age of 2 years, which was felt to be an important developmental milestone and previous research indicates that “picky eating” peaks 2 years of age [20].

## Results

Of the total of 1197 questionnaires submitted, 293 were excluded (incomplete submissions *n* = 243, Pedi-EAT-10 score > 3 *n* = 50), leaving 904 responses for analysis. There were no statistically significant differences on any demographic variables between included and excluded cases. Demographic details of participants are outlined in Table 1. Details regarding specific location of participants within the UK are provided in supplementary material. The median (IQR) age of children included was 5.5 years (1–7 years).

**Table 1:** Participant demographic, social and medical history characteristics

Demographic, social and medical history characteristics	Number of participants (%)
*Household members*	
My child(ren)	38 (4.2)
My child(ren) and partner	841 (93)
My child(ren) and extended family/friends	7 (0.8)
My child(ren), partner and extended family/friends	18 (2)
*Relationship to child*	
Parent	903 (99.9)
Grandparent	1 (0.1)
*Parent gender*	
Male	96 (10.6)
Female	801 (88.6)
Non-binary	5 (0.6)
Prefer not to say	2 (0.2)
*Parent ethnicity*	
White	787 (87.1)
Asian/Asian-British	42 (4.6)
Black/Black-British	11 (1.2)
Mixed	46 (5.1)
Prefer not to say	4 (0.4)
Not listed	14 (1.5)
*Employment status*	
Employed for wages	761 (84.1)
Self-employed	65 (7.2)
Not employed	11 (1.2)
Homemaker	51 (5.6)
Student	16 (1.8)
*Parent age*	
18–29	60 (6.6)
30–39	575 (63.6)
40–59	269 (4.8)
*Number of children in household*	
1	413 (45.7)
2	406 (44.9)
3 or more	85 (9.4)
*Child gender*	
Male	453 (50.8)
Female	445 (49.2)
*Child age (uncorrected for prematurity)*	
<2 years	240 (26.5)
≥2 years	664 (73,5)
*Gestational age*	
Term	862 (95.3)
33–36 weeks	33 (3.6)
≤32 weeks	9 (1.0)
*Food allergy*	
No	821 (90.8)
Yes	83 (9.2)
*History of GOR*	
No	764 (84.5)
Yes	134 (14.8)
Unknown	6 (0.7)
*History of professional support for feeding*	
No	809 (89.5)
Yes	95 (10.5)
*Parent perceived feeding difficulty*	
No	892 (98.6)
Yes	12 (1.4)

GOR = gastro-oesophageal reflux.

### Summary of Responses

Frequency of responses for each FS-IS question are provided in supplementary material. Mean, median and spread are provided for the whole scale and subscales scores in Table 2. Using the summed average (mean) scoring system, total and subscale scores were near floor level. Standard deviations were all small, indicating a narrow range of data. The FS-IS has a cumulative score range of 18–90. Yet, the median total score in our cohort was 20, with an IQR of just 3.

**Table 2:** Mean and median FS-IS cumulative and average scoring systems

	Average scoring Mean (SD)	Average scoring Median (IQR)	Cumulative scoring Mean (SD)	Cumulative scoring Median (IQR)
Whole scale score	1.17 (0.20)	1.11 (1.06, 1.22)	20.98 (3.66)	20.00 (19.00, 22.00)
Activity subscale	1.15 (0.36)	1.00 (1.00, 1.20)	5.77 (1.82)	5.00 (5.00, 6.00)
Worry subscale	1.28 (0.29)	1.29 (1.14, 1.43)	8.95 (2.02)	9.00 (8.00, 10.00)
Feeding subscale	1.04 (0.15)	1.00 (1.00, 1.00)	6.24 (0.87)	6.00 (6.00, 6.00)

Possible FS-IS cumulative scoring ranges: total score 18–90, activity subscale 5–25, worry subscale 7–35, feeding subscale 6–30. Possible FS-IS average scoring range 1–5.

### Impact of Demographic and Medical History Variables On FS-IS Total Score

Variables with significant differences in median scores (parent age, child age, number of children, food allergy, history of GOR, history of professional support for feeding, parent perceived feeding difficulty) and determined as logical variables were included in a quantile regression to evaluate the predictive value of these variables for the 75th centile whole scale cumulative FS-IS score. Test assumptions were met. Table 3 shows the factors contributing to the model. The 75th centile was on average 24 points higher for parents who perceived their child to have a feeding difficulty compared to those who did not. Families with more than one child in the household had a 3 point lower score in the 75th centile compared to those with only 1 child. Children over 2 years of age had a 3 point lower score compared to those under 2. Parents aged over 40 had a 3 point lower score compared to those aged under 30. Those with a history of receiving professional support for feeding and those with a food allergy had a 3 point higher score than those without. The overall model explained 10.8% of score variation at the 75th centile.

**Table 3:** Quantile regression for 75th centile feeding-swallowing impact survey whole scale score

	Estimated 75th centile	Lower 95% CI	Upper 95% CI	*p* value
*Model*				
Parent age 30–39	−2	−4.14	0.14	0.07
Parent age 40–49	−3	−5.29	− 0.71	0.01
2 or more child household	−3	−4.09	−1.90	< 0.001
Child over 2 years	−3	−4.22	−4.22	< 0.001
Presence of food allergy	3	1.10	4.90	0.002
History of GOR	0	−1.52	1.52	1.00
History of professional support	3	0.1.19	4.81	0.001
Parent perceived difficulty	24	19.36	28.64	< 0.001

CI = confidence interval, GOR = gastro-oesophageal reflux

### Reference Values

Percentile ranks for FS-IS total scores (rounded to the nearest whole number) are provided in Table 4.

**Table 4:** Percentile ranks for cumulative, whole scale FS-IS scores for children aged 6 months - <2 years and children aged 2–11 years

Percentile	FSIS whole scale score 6 months-11 years (*n* = 904)	FS-IS whole scale score 6 months-<2 years (*n* = 240)	FS-IS whole scale score 2–11 years (*n* = 664)
5	18	18	18
25	19	19	18
50	20	21	20
75	22	24	21
95	29	34	26

## Discussion

This study provides normative reference values for the FS-IS from a large UK population of parents of children aged 6 months-11 years without known or suspected feeding/swallowing difficulties. Our data indicate that despite mealtimes often being a source of worry for parents, when a child does not have feeding or swallowing difficulties, quality of life is largely unaffected. Our findings suggest that use of cumulative scores rather than mean scores improves clinical utility of the FS-IS by more clearly capturing score differences and generating clinically applicable percentile ranks. This aids interpretation of FS-IS scores for those *with* PFD, providing evidence from which to determine the extent to which feeding-related QOL is impacted. Identifying families in which QOL is adversely impacted facilitates onward referral or signposting for additional psychological support and identification of therapy goals.

Two studies have included parents of children without feeding disorders as control subjects. Serel Arslan used a Turkish adaptation of the tool to evaluate 31 mothers of children aged 18–29 months, reporting a mean total score of 1.53 (SD 0.47) [9]. Madhoun and colleagues reported mean total FS-IS score of 1.52 (SD 0.67) for 30 mothers of children aged 9–90 days [15]. Both are slightly higher than our sample mean. The cohort in the Madhoun et al. study were under three months, which may explain the slightly higher mean scores- reflecting the challenge of establishing feeding early in life and the impact that it has on mothers. The higher scores in the Serel Arslan cohort may reflect differences in the translated version of the tool, cultural differences or be reflective of the smaller sample size and greater data spread.

Twelve parents reported that their child had more difficulties than others of the same age, despite not meeting the threshold for potential difficulty on the pedi-EAT-10. This suggests that *perception* of feeding problems may influence scoring. When presented with the same mealtime characteristics, factors such as parenting self-efficacy, both general and mealtime specific and underlying parent anxiety, have been shown to be related to challenging mealtimes in typically developing children [30–32]. Such aspects of parent-child interaction may serve as important modulators in FS-IS scoring but were unexplored in our study and could be important areas for further research to guide development of interventions targeting feeding-related QOL.

### Limitations

This study recruited a large sample of participants from across the UK. Specific efforts were made to target populations that traditionally do not engage in this type of research. However, despite this the profile of responders was largely from the London and South-East region, female, white, employed and living with a partner and the potential for bias must be acknowledged. Comparing our cohort to UK regional populations acquired in 2021 Census data, our sample was slightly over-representative of white and mixed ethnicity and slightly under-representative of other ethnicities [33]. Additionally, the questionnaire was only available in English, excluding those without sufficient English language skills from participating. The study also relied on digital competence and internet access in recruitment and questionnaire completion. The digital nature of the study may have influenced participation to those most confident or comfortable with technology. Therefore, cautious use of reference values when the tool is used with under- or unrepresented groups is warranted.

The FS-IS was initially designed to be completed using a paper version of the tool. Our study used an electronic version. Online tools are becoming increasingly popular, particularly with the rise in telehealth and online research methods [34]. The impact of online completion, compared to a paper version is unclear.

The study aimed to generate reference values from a community population of parents of children without known PFD. As no brief screen for PFD exists for children aged 6 months-11 years, the Pedi-EAT-10 was chosen as the best fit screen for swallowing/feeding difficulties [25]. It is therefore possible that some parents of children with PFD could have been included in the reference sample and some without PFD excluded.

### Conclusions

This study demonstrates that despite “feeding difficulties” being commonly reported in typically developing children, feeding-related QOL is rarely impacted for these parents. We have generated reference values (mean, median and percentile rank) for the FS-IS from which clinical and research populations can be compared. These data allow clinicians and researchers to understand the extent to which feeding-related QOL is impacted. This facilitates identification of parents who may require further evaluation and support to lessen the impact of feeding-related issues on QOL.

## Electronic Supplementary Material

Below is the link to the electronic supplementary material.

Supplementary Material 1

Supplementary Material 2

## Data Availability

Raw data files from which analysis was undertaken can be found here: Feeding swallowing impact survey UK reference values raw data (ucl.ac.uk).
