# Supplementary material for: The Feeding-swallowing Impact Survey: Reference Values from a UK Sample of Parents of Children Without a Known or Suspected Feeding Disorder
Source: Dysphagia. 2025 Jun 5;40(6):1479–85. doi: 10.1007/s00455-025-10845-z (PMC12662897; doi:10.1007/s00455-025-10845-z)
Supplement: Supplementary file 1 — Supplementary Material 1 [file 455_2025_10845_MOESM1_ESM.pdf]

# Demographics

Thank you for taking part in this survey!

This page will ask questions about you and your child's demographic information.

## A little bit about you

| | |
|---|---|
| Where do you live? | ◯ England - London<br>◯ England - South East<br>◯ England - South West<br>◯ England - West Midlands<br>◯ England - East Midlands<br>◯ England - Yorkshire and the Humber<br>◯ England - North West<br>◯ England - North East<br>◯ Scotland<br>◯ Wales<br>◯ Northern Ireland<br>◯ Not listed - please state |
| Please specify where you live: | __________________________________ |
| Who do you live with? | ◯ My child(ren)<br>◯ My child(ren) and partner<br>◯ My child(ren) and extended family/friends<br>◯ My child(ren), partner and extended family/friends<br>◯ Other |
| Please specify who you live with: | __________________________________ |
| What is your relationship to the child? | ◯ Parent<br>◯ Grandparent<br>◯ Legal guardian<br>◯ Other |
| Please specify your relationship to the child: | __________________________________ |
| How would you describe your gender? | ◯ Male<br>◯ Female<br>◯ Non-binary<br>◯ I'd prefer not to say<br>◯ Other |
| Please specify your gender: | __________________________________ |
| How would you describe your ethnicity? | ◯ White<br>◯ Asian / Asian-British<br>◯ Black / Black-British<br>◯ Mixed<br>◯ I'd rather not say<br>◯ Other |
| Please specify your ethnicity: | __________________________________ |

| | |
|---|---|
| What type of work do you do? | ◯ Employed for wages<br>◯ Self-employed<br>◯ Out of work and looking for work<br>◯ Out of work but not currently looking for work<br>◯ Homemaker<br>◯ Student<br>◯ Retired<br>◯ Unable to work<br>◯ I'd rather not say |
| How old are you? | ◯ 18-20<br>◯ 21-29<br>◯ 30-39<br>◯ 40-49<br>◯ 50-59<br>◯ 60+ |
| How many children do you care for in your household? | ◯ 1<br>◯ 2<br>◯ 3+ |

**A little bit about your child**

**Please think about one of the children you care for in your household when answering the rest of the questions. The child you are answering about should be between the ages of 6 months and 11 years.**

| | |
|---|---|
| Is the child you are answering for your...<br><br>(If answering for a twin/triplet/etc., please select according to birth order) | ◯ Eldest child<br>◯ Youngest child |
| Is the child you are answering for your...<br><br>(If answering for a twin/triplet/etc., please select according to birth order) | ◯ Eldest child<br>◯ Middle child<br>◯ Youngest child |
| Is the child you are answering for a twin/triplet/etc.? | ◯ Yes<br>◯ No |
| What is this child's gender? | ◯ Male<br>◯ Female<br>◯ Non-binary<br>◯ I'd prefer not to say<br>◯ Other |
| Please specify your child's gender: | __________________________________ |

# FS-IS

This is the final part of the survey.

Please answer the following questions from the Feeding-Swallowing Impact Survey.

| | |
|---|---|
| How old is your child? | ◯ 6-11 months<br>◯ 1 year<br>◯ 2 years<br>◯ 3 years<br>◯ 4 years<br>◯ 5 years<br>◯ 6 years<br>◯ 7 years<br>◯ 8 years<br>◯ 9 years<br>◯ 10 years<br>◯ 11 years |
| Was your child born early (prematurely)? | ◯ No<br>◯ 1-3 weeks early (37-39 weeks)<br>◯ 4-7 weeks early (33-36 weeks)<br>◯ 8-12 weeks early (28-32 weeks)<br>◯ 13+ weeks early (22-27 weeks) |
| Does your child have any food allergies? | ◯ Yes<br>◯ No |
| Please let us know what your child is allergic to: | __________________________________ |
| Has your child ever needed any treatment for reflux (also known as gastro-oesophageal reflux or GOR)? | ◯ Yes<br>◯ No<br>◯ I don't know |
| Has your child received any treatment for reflux in the LAST MONTH? | ◯ Yes<br>◯ No |
| Other than a midwife, health visitor or lactation consultant, have you ever seen any health professional about your child's feeding, eating or drinking? | ◯ Yes<br>◯ No |
| Do you feel that your child has more difficulties eating and drinking compared to others their age? | ◯ Yes<br>◯ No |
| Please specify these difficulties: | __________________________________ |

**Thinking about your child's eating and drinking over the LAST WEEK, how much of a problem have you found these issues?**

| | 0 No problem | 1 | 2 | 3 | 4 Severe problem |
|---|---|---|---|---|---|
| My child does not gain weight due to his/her swallowing (problem) | ○ | ○ | ○ | ○ | ○ |
| The swallowing (problem) of my child interferes with our ability to go out for meals | ○ | ○ | ○ | ○ | ○ |
| Swallowing liquids takes extra effort for my child | ○ | ○ | ○ | ○ | ○ |
| Swallowing solids takes extra effort for my child | ○ | ○ | ○ | ○ | ○ |
| My child gags during swallowing | ○ | ○ | ○ | ○ | ○ |
| My child acts like he/she is in pain while swallowing | ○ | ○ | ○ | ○ | ○ |
| My child does not want to eat | ○ | ○ | ○ | ○ | ○ |
| Food sticks in my child's throat and my child chokes while eating | ○ | ○ | ○ | ○ | ○ |
| My child coughs while eating | ○ | ○ | ○ | ○ | ○ |
| Swallowing is stressful for my child | ○ | ○ | ○ | ○ | ○ |

**The next questions ask about how your child's feeding might have affected you as a parent. When answering these questions, please think about how you've felt in the last ONE MONTH. How often have you felt...**

| | 1 Never | 2 | 3 | 4 | 5 Almost always |
|---|---|---|---|---|---|
| It is hard for me to do my job, go to school, or work around the house | ○ | ○ | ○ | ○ | ○ |
| It is hard for me to get help from others because they are scared to feed or take care of my child | ○ | ○ | ○ | ○ | ○ |
| It is hard for me to leave my child because I am scared to have other people feed or take care of my child | ○ | ○ | ○ | ○ | ○ |
| It is hard for my family to make plans or go out to eat | ○ | ○ | ○ | ○ | ○ |
| I am too tired to do the things I want or need to do | ○ | ○ | ○ | ○ | ○ |

**When answering think about how you've felt in the last ONE MONTH. How often have you felt...**

| | 1 Never | 2 | 3 | 4 | 5 Almost always |
|---|---|---|---|---|---|
| I worry about my child's general health | ○ | ○ | ○ | ○ | ○ |
| I worry that my child does not get enough to eat or drink | ○ | ○ | ○ | ○ | ○ |
| I worry about how others will react to my child's feeding/swallowing (problems) | ○ | ○ | ○ | ○ | ○ |
| I worry about how my child breathes when feeding or whether my child will choke | ○ | ○ | ○ | ○ | ○ |
| I worry that my child will never eat or drink like other children | ○ | ○ | ○ | ○ | ○ |
| I worry about whether I am doing enough to help with my child's feeding/swallowing (problems) | ○ | ○ | ○ | ○ | ○ |
| I worry about how my child's feeding/swallowing (problems) affect others in my family | ○ | ○ | ○ | ○ | ○ |

**When answering think about how you've felt in the last ONE MONTH. How often have you felt...**

| | 1 Never | 2 | 3 | 4 | 5 Almost always |
|---|---|---|---|---|---|
| It is hard to feed my child because it takes a long time to prepare liquids or foods the "right" way | ○ | ○ | ○ | ○ | ○ |
| It is hard to feed my child because I don't know how to prepare liquids or foods | ○ | ○ | ○ | ○ | ○ |
| It is hard to feed my child because others give my child liquids or foods that are not allowed | ○ | ○ | ○ | ○ | ○ |
| It is hard to feed my child because I don't know how long these feeding/swallowing (problems) will last | ○ | ○ | ○ | ○ | ○ |

| | | | | | |
|---|---|---|---|---|---|
| It is hard to feed my child because family members or professionals have different opinions about how to take care of my child's feeding/swallowing | ◯ | ◯ | ◯ | ◯ | ◯ |
| It is hard to feed my child because I do not get enough information about how to get my child to eat or drink like other children | ◯ | ◯ | ◯ | ◯ | ◯ |

## Anything else?

Optionally, is there anything else you would like to tell us about your child's feeding or it's impact on you or your family? ________________________________________
