# Supplementary material for: The Feeding-swallowing Impact Survey: Reference Values from a UK Sample of Parents of Children Without a Known or Suspected Feeding Disorder
Source: Dysphagia. 2025 Jun 5;40(6):1479–85. doi: 10.1007/s00455-025-10845-z (PMC12662897; doi:10.1007/s00455-025-10845-z)
Supplement: Supplementary file 2 — Supplementary Material 2 [file 455_2025_10845_MOESM2_ESM.docx]

Feeding-swallowing impact survey: frequency of responses (whole cohort, n=904)

|  | Question rating | | | | |
| --- | --- | --- | --- | --- | --- |
|  | **1**  **Never**  **%** | **2**  **%** | **3**  **%** | **4**  **%** | **5**  **Almost always**  **%** |
| 1. It is hard for me to do my job, go to school, or work around the house | 89.9 | 7 | 2.2 | 0.7 | 0.2 |
| 2. It is hard for me to get help from others because they are scared to feed or take care of my child | 96.6 | 2.4 | 0.4 | 0.4 | 0.1 |
| 3. It is hard for me to leave my child because I am scared to have other people feed or take care of my child | 91.8 | 5.1 | 1.8 | 0.8 | 0.6 |
| 4. It is hard for my family to make plans or go out to eat | 90.3 | 6.7 | 2.3 | 0.4 | 0.2 |
| 5. I am too tired to do the things I want or need to do | 79.3 | 12.7 | 5 | 1.8 | 1.2 |
| 6. I worry about my child’s general health | 32.5 | 44.9 | 18.6 | 2.8 | 1.3 |
| 7. I worry that my child does not get enough to eat or drink | 51.9 | 33.5 | 11.5 | 2.4 | 0.7 |
| 8. I worry about how others will react to my child’s feeding/swallowing problems | 97.3 | 2.2 | 0.3 | 0 | 0.1 |
| 9. I worry about how my child breaths when feeding or whether my child will choke | 89.1 | 8 | 2.3 | 0.4 | 0.2 |
| 10. I worry that my child will never eat or drink like other children | 94.4 | 3.3 | 1.3 | 0.7 | 0.3 |
| 11. I worry about whether I am doing enough to help with my child’s feeding/swallowing problems | 95.6 | 3.1 | 1 | 0.3 | 0 |
| 12. I worry about how my child’s feeding/swallowing (problems) affect others in my family | 98.9 | 0.7 | 0.3 | 0.1 | 0 |
| 13. It is hard to feed my child because it takes a long time to prepare liquids and foods the “right” way | 91.4 | 7.2 | 1.3 | 0.1 | 0 |
| 14. It is hard to feed my child because I don’t know how to prepare liquids or foods | 97.9 | 1.8 | 0.3 | 0 | 0 |
| 15. It is hard to feed my child because others give my child liquids or food that they are not allowed | 96.9 | 2.2 | 0.6 | 0.3 | 0 |
| 16. It is hard to feed my child because I don’t know how long these problems will last | 98.2 | 1.5 | 0.1 | 0.1 | 0 |
| 17. It is hard to feed my child because family members or professionals have different opinions about how to take care of my child’s feeding/swallowing (problems) | 97.1 | 2.3 | 0.4 | 0.1 | 0 |
| 18. It is hard to feed my child because I do not get enough information about how to get my child to eat or drink like other children | 97.6 | 1.4 | 0.9 | 0.1 | 0 |
